# Clinical characteristics and long-term prognosis of spontaneous coronary artery dissection: A single-center Chinese experience

**DOI:** 10.12669/pjms.35.1.321

**Published:** 2019

**Authors:** Xintian Liu, Chengyi Xu, Chengwei Liu, Xi Su

**Affiliations:** 1 Xintian Liu, Department of Cardiology, Wuhan Asia Heart Hospital, Wuhan University of Science and Technology, Wuhan, China; 2 ChengyiXu, Department of Cardiology, Wuhan Asia Heart Hospital, Wuhan University of Science and Technology, Wuhan, China; 3 Chengwei Liu, Department of Cardiology, Wuhan Asia Heart Hospital, Wuhan University of Science and Technology, Wuhan, China; 4 Xi Su, Department of Cardiology, Wuhan Asia Heart Hospital, Wuhan University of Science and Technology, Wuhan, China

**Keywords:** Acute coronary syndrome, Coronary angiography, Coronary artery bypass grafting, Coronary artery disease, Percutaneous coronary intervention, Spontaneous coronary artery dissection

## Abstract

**Background and Objective:**

Spontaneous coronary artery dissection (SCD) remains a rare and important cause of coronary artery disease (CAD). The purpose of this study was to describe the clinical and angiographic features in SCD and to evaluate the treatment and long-term prognosis of this condition in China.

**Methods:**

This retrospective cohort study included 118 Chinese patients with SCD confirmed by coronary angiography. Clinical and angiographic features, treatment modalities and outcomes of SCD were estimated.

**Results:**

The overall prevalence of SCD was 0.15%. Age was 57 ± 10 years; 86% patients were men; 75% presented with acute coronary syndrome (ACS); 72% had concomitant atherosclerotic CAD. SCD often affected right coronary artery (RCA) and caused a short dissection (< 20mm). A conservative therapy was used in 28% of patients and revascularization in 72% (percutaneous coronary intervention [PCI] 57%; coronary artery bypass grafting [CABG] 15%). Only one patient died during hospitalization due to multiple organ failure after CABG. During a median follow-up of 43 months (range, 1 - 158 months), 32 patients had a new-onset ACS, 9 received revascularization (7 PCI and 2 CABG), and 8 died. The Kaplan-Meier estimated 12-year rates of freedom from cardiac death and ACS were both higher in revascularization versus conservative therapy (78% versus 57%; P = 0.023; 48% versus 25%, P = 0.014). No significant difference was found in freedom from revascularization between the two therapies.

**Conclusions:**

In China, SCD was usually associated with atherosclerosis and predominantly affected male population. SCD often affected RCA and caused a short dissection. In-hospital mortality rate was low regardless of therapeutic strategy. However, a significantly better long-term prognosis was observed in the revascularization compared with conservative therapy.

## INTRODUCTION

Spontaneous coronary artery dissection (SCD), a separation of the arterial wall with the creation of a false lumen that is not related to medical instrumentation or trauma, remains an infrequent cause of acute coronary syndrome (ACS).^1^ So far, fewer than 1500 cases have been reported since the first case reported in 1931.^2^ However, the diagnosis of SCD has apparently increased recently, probably due to the increased use of coronary angiography and intracoronary imaging techniques like intravascular ultrasound (IVUS) and optical coherence tomography (OCT).^3^ Publications of several large studies of SCD that have been reported in recent years are helpful in elucidating the underlying etiology and angiographic features and improving the management and outcomes of this condition. However, most of patients in these studies are from developed countries and not reflect the reality of patients with SCD in China. The purpose of this study was to describe the clinical and angiographic features in SCD and to evaluate the treatment and long-term prognosis of this condition in China.

## METHODS

### Patients

Through January 2003 to December 2015, a total of 76,359 primary coronary angiographies were performed in our center. Of which, 531 potential patients were yielded by searching relevant key words in hospital database, including dissection, filling defect, intimal tear or intimal flap. The definition of SCD in this study was the presence of a longitudinal radiolucent linear image in at least two orthogonal projections proven on coronary angiography and confirmed by two experienced interventional cardiologists. Iatrogenic and traumatic SCD and previous coronary intervention were excluded. Finally, the diagnosis of SCD was identified in 118 patients. This retrospective cohort study was approved by the Ethics Committee of Wuhan Asia Heart Hospital and the need for individual patient consent was waived.

### Data Collection and Definitions

Baseline risk factors, clinical presentation, treatment modality and in-hospital events were collected via medical records. Long-term outcomes were gained via clinical visits, telephone follow-up, or medical record review in the case of readmission. For deceased patients, information was obtained by telephone from the immediate family or contact with local police. The primary long-term end points included cardiac death, ACS (including ST-segment elevation myocardial infarction [STEMI], non-ST-segment elevation myocardial infarction [NSTEMI] and unstable angina pectoris [UAP]) and revascularization.

The National, Heart, Lung, and Blood Institute (NHLBI) classification and the Thrombolysis in Myocardial Infarction (TIMI) flow grade classification were used to characterize the SCD lesions.^4^,^5^ The coronary segment involved with SCD was classified by the Bypass Angioplasty Revascularization Investigation (BARI) classification.^6^ The dissection length was divided into two groups: short (< 20mm) and long (≥ 20mm). Atherosclerotic coronary artery disease (CAD) was defined as at least one coronary lesion (different from the SCD lesion) with a diameter stenosis ≥ 50% on visual assessment.

Successful percutaneous coronary intervention (PCI) was defined as TIMI flow grade = 3 after balloon angioplasty or stent implantation. Unsuccessful PCI was defined as TIMI flow grade < 3 or extension of dissection after PCI. Revascularization included PCI and coronary artery bypass grafting (CABG).

### Statistical Analysis

Categorical data were presented as frequency and percentage and continuous data as mean ± SD. The chi-square or Fisher exact tests were performed for categorical data. The Kaplan-Meier curves were plotted by time-to-event data and compared by the log-rank tests. All analyses were performed with SPSS software. A two-side value of P < 0.05 was considered statistically significant.

## RESULTS

**Fig.1:**
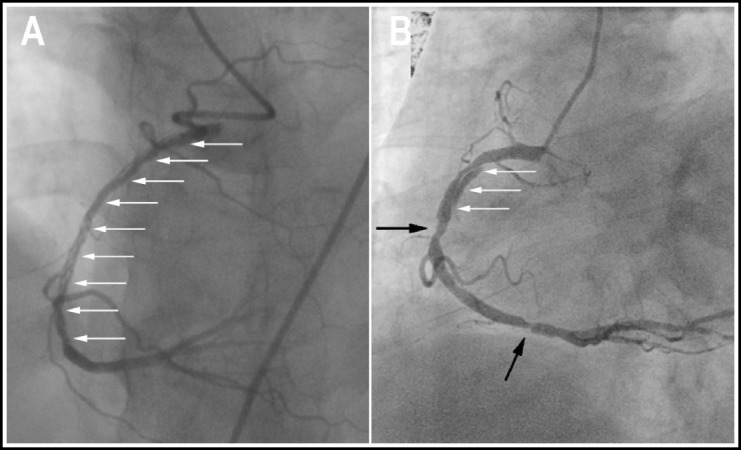
Two types of SCD. One is non-atherosclerotic (A) and the other is atherosclerotic (B). White arrows denote the angiographic intimal flap. Black arrows denote atherosclerotic lesions. SCD: Spontaneous coronary artery dissection.

The overall prevalence of SCD in the present study was 0.15% (118 per 76359 subjects). The baseline of patients with SCD is described in Table-I. The mean age was 57.4 ± 10.3 (range, 32-82) years and 102 (86.4%) patients were men. SCD patients had a relatively high prevalence of conventional cardiovascular risk factors. No one in the study presented in the postpartum period or had a drug addiction history. A sizable proportion of SCD patients had a history of stroke (13.6%) and old myocardial infarction (33.1%). There were 88 (74.6%), 11 (9.3%) and 10 (8.5%) patients presented with an ACS, stable angina and heart failure, respectively. The remaining 9 (7.6%) patients were asymptomatic. Notably, Most SCD patients (72.0%) had atherosclerotic CAD (Fig.1).

**Table-I:** Baseline Characteristics of SCD patients.

Baseline Characteristics	n = 118
Age, years	57.4 ± 10.3
*Gender*	
Men	102 (86.4%)
Women	16 (13.6%)
Body mass index, Kg/m^2^	26.0±3.2
Smoking	82 (69.5%)
Alcohol drinking	54 (45.8%)
Hypertension	74 (62.7%)
Diabetes mellitus	44 (37.3%)
Hyperlipidemia	51 (43.2%)
Previous stroke	16 (13.6%)
OMI	39 (33.1%)
*Clinical presentation*	
STEMI	28 (23.7%)
NSTEMI	28 (23.7%)
Unstable angina	32 (27.1%)
Stable angina	11 (9.3%)
Heart failure	10 (8.5%)
Asymptomatic	9 (7.6%)
Left ventricular end diastolic diameter, cm	5.2 ± 0.7
Left ventricular ejection fraction, %	50.1 ± 9.4
Regional ventricular wall motion abnormality	65 (55.1%)
Ventricular aneurysm	13 (11.0%)
Concomitant atherosclerotic coronary artery disease	85 (72.0%)

Values are mean ± SD or n (%). SCD: Spontaneous coronary artery dissection, OMI: old myocardial infarction, STEMI: St-segment elevation myocardial infarction, NSTEMI: Non-st-segment elevation myocardial infarction.

The main coronary angiographic characteristics are reported in Table-II. The majority (97.5%) had only 1 coronary artery dissection. Involvement of at least 2 major coronary branches was seen in three patients (two showed two-vessel dissection and one showed 3-vessel dissection). SCD lesions were often located in the right coronary artery (RCA) and proximal segments of dissected-vessel. Most SCD lesions (76.2%) were short (length < 20 mm), especially in patients receiving PCI (85.3%). The anterior descending artery (LAD) and triple-vessel lesions (including SCD and atherosclerotic CAD) were more frequent in CABG, compared with conservative and PCI.

**Table-II:** Angiographic Findings.

	Total (n=118)	Conservative (n=33)	PCI (n=67)	CABG (n=18)	P
*SCD information*					
Multi-vessel dissection	3 (2.5)	1 (3.0)	1 (1.5)	1 (5.6)	0.610
SCD lesions	122	34	68	20	
SCD distribution					0.992
LM	7 (5.7%)	2 (5.9%)	4 (5.9%)	1 (5.0%)	
LAD	30 (24.6%)	8 (23.5%)	16 (23.5%)	6 (30.0%)	
LCX	6 (4.9%)	1 (2.9%)	4 (5.9%)	1 (5.0%)	
RCA	79 (64.8%)	23 (67.6%)	44 (64.7%)	12 (60.0%)	
SCD segment					0.460
Proximal	58 (47.5%)	20 (58.8%)	28 (41.2%)	10 (50.0%)	
Mid	37 (30.3%)	8 (23.5%)	22 (32.4%)	7 (35.0%)	
Distal	27 (22.2%)	6 (17.6%)	18 (26.5%)	3 (15.0%)	
SCD lesion length					0.025
< 20 mm	93 (76.2%)	23 (67.6%)	58 (85.3%)	12 (60.0%)	
≥ 20 mm	29 (23.8%)	11 (32.4%)	10 (14.7%)	8 (40.0%)	
NHLBI classification*					0.018
A-B-C	58 (47.5%)	12 (35.3%)	40 (58.8%)	6 (30.0%)	
D-E-F	64 (52.5%)	22 (64.7%)	28 (41.2%)	14 (70.0%)	
SCD TIMI flow					0.898
0-2	29 (23.8%)	8 (23.5%)	17 (25.0%)	4 (20.0%)	
3	93 (76.2%)	26 (76.5%)	51 (75.0%)	16 (80.0%)	

**Total lesions information (including SCD and concomitant atherosclerotic coronary artery disease)**					

Triple-vessel lesion	42 (35.6)	9 (27.3)	22 (32.8)	11 (61.1)	0.042
*Distribution of total lesions*					
LM	9 (7.6)	3 (9.1)	5 (7.5)	1 (5.6)	0.899
LAD	82 (69.5)	21 (63.6)	44 (65.7)	17 (94.4)	0.043
LCX	65 (55.1)	15 (45.5)	38 (56.7)	12 (66.7)	0.319
RCA	90 (76.3)	25 (75.8)	51 (76.1)	14 (77.8)	0.986

Values are n (%).

* Type-A: radiolucent area within the lumen, minimal/no persistence of contrast, Type-B: parallel double lumen separated by a radiolucent area with minimal/no persistence of contrast, Type-C: persistent presence of contrast outside the lumen, Type-D: spiral luminal filling defect, Type-E: dissection with persistent filling defect, Type-F: dissection with total coronary occlusion. SCD: Spontaneous Coronary artery Dissection, PCI: percutaneous coronary intervention, CABG: coronary artery bypass grafting, LM: left main trunk artery, LAD: left anterior descending artery, LCX: left circumflex artery, RCA: right coronary artery, TIMI: thrombolysis in myocardial infarction.

**Fig.2:**
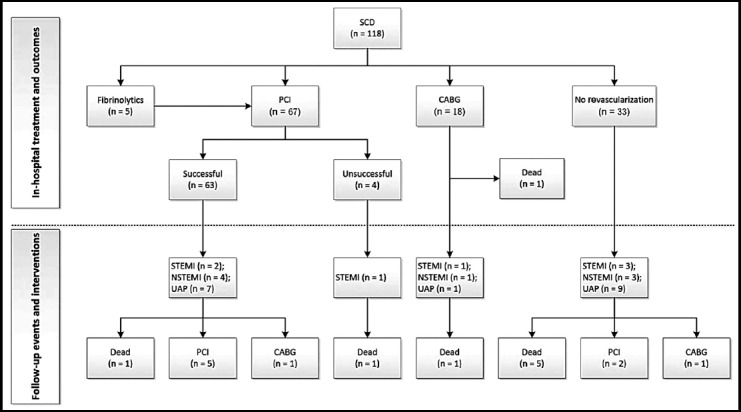
Flow chart showing management and outcomes for patients with SCD. SCD: Spontaneous coronary artery dissection; CABG: Coronary artery bypass surgery; PCI: Percutaneous coronary intervention; STEMI: st-segment elevation myocardial infarction; NSTEMI: Non-st-segment elevation myocardial infarction; UAP: unstable angina pectoris.

Most patients with SCD (85/118; 72.0%) underwent PCI or CABG during hospitalization (Fig.2). Five patients received thrombolytic therapy before PCI. Of 67 PCIs, 29 were used to address dissection lesions only and 38 to address both dissection and atherosclerotic lesions. The majority (63/67, 94%) of PCI were successful. Reasons for technical failure in four PCI were guide wire (n=2) or balloon (n=1) failing to cross the SCD lesions and worse flow after PCI (n=1). Eighteen patients underwent CABG and one died due to the multiple organ failure after surgery during hospitalization. Dual-antiplatelet and anticoagulant therapy were used in 93.2% and 76.3% of patients, respectively.

Clinical follow-up (median, 43 months; interquartile range, 25 - 75 months; range, 1 - 158 months) was obtained in all 117 discharged patients. Overall, 32 (27.4%) of discharged patients had a new-onset ACS (7 STEMI, 8 NSTEMI and 17 UAP), 9 (7.7%) received revascularization (7 PCI and two CABG), and 8 (6.8%) had died at 12 years. Detail follow-up events and interventions based on therapeutic strategy are also showed in Fig.2.

Repeated coronary angiography was performed in 12 of 32 patients with a new-onset ACS during follow-up. In four patients initially receiving conservative treatment, their six dissected vessels (2 single- and two double-vessel dissections) demonstrated persistent dissection. Two of them were treated with PCI. One PCI was successful and the other was unsuccessful due to balloon failing to cross the SCD lesion. Of 8 patients initially being treated with PCI treatment, seven had in-stent restenosis (5 at dissected lesions, two at atherosclerotic lesions) and one had stent thrombosis at SCD lesion at follow-up. Five of 8 patients received re-PCI (four for dissection lesions and one for both dissection and atherosclerotic lesion) and all five PCIs were successful. Overall, Most of PCI procedures (53/67, 79.1%) had excellent results, without need for repeat revascularization. There was no follow-up coronary angiography in patients with CABG.

**Fig.3:**
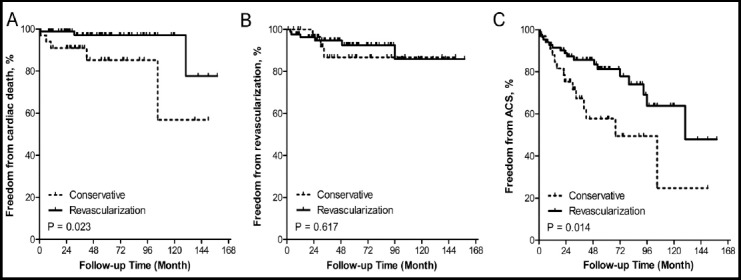
Long-term follow-up for SCD patients according to treatment strategy. (A) Freedom from cardiac death (B) Freedom from revascularization (C) Freedom from ACS SCD: Spontaneous coronary artery dissection; ACS: Acute coronary syndrome.

Long-term outcomes of SCD patients are presented in Fig.3. Freedom from cardiac death was higher in revascularization than conservative therapy at 12 years (77.6% versus 56.8%, P=0.023, Fig.3A). No difference was found in freedom from revascularization between the two therapies (7.1% versus 9.1%, P = 0.617, Fig.3B). The Kaplan-Meier estimated rate of freedom from ACS at 12 years was significantly higher in revascularization than conservative therapy (47.8% versus 24.7%, P = 0.014, Fig.3C).

## DISCUSSION

To the best of our knowledge, this respectively cohort study reports the largest series of SCD patients and provides the longest clinical follow-up in China. In this study, we found that SCD was often associated with atherosclerosis and presented with an ACS, with a high prevalence of conventional cardiovascular risk factors. Moreover, SCD often affected RCA and proximal vessel segment and caused a short dissection. The technical success rate of revascularization was satisfactory. In-hospital mortality was low regardless of treatment strategy. During follow-up, a significantly better long-term outcome was observed in the revascularization versus conservative therapy.

Previous series have reported the SCD detection in 0.07% to 1.1% of all coronary angiograms performed.^7^ The prevalence of SCD in this study is 0.15% (118 per 76359 subjects), consistent with these series. Pathologically, SCD can be broadly classified into atherosclerotic (A-SCD) and non-atherosclerotic (NA-SCD) SCD. NA-SCD tends to affect young women without or with a low incidence of cardiovascular risk factors^8^, while A-SCD tends to influence old men with a high incidence of cardiovascular risk factors.^9^ A-SCD accounts for about 40% of SCD in a western study.^10^ However, this figure is significantly higher in Asian countries: 72% in our study and 92% in a Japanese study.^11^ Therefore, unlike NA-SCD being the leading form of SCD in western countries, A-SCD may be the leading form of SCD in China.

There is a wide spectrum of clinical presentations of SCD due to a variety of extent and the flow limiting severity of dissection and the main presentation is ACS.^7^ There are similar results in our study. Notably, 8.5% patients in the study have heart failure on admission. Yet, this figure has not been reported in other studies from developed countries. This is probably due to the prevalence of old myocardial infarction in this study (33%) being much higher than other studies (1.8% - 5.3%).^12^,^13^ The main reason behind it might be that China is still a developing country and the public health consciousness and the medical treatment system in China are not as good as those in developed countries.

Medical treatment of SCD in this study is similar to the treatment of ACS, including antiplatelet, anticoagulant and anti-ischemic therapy. Yet, the role of thrombolytic is debated. Previous NA-SCD studies suggested that thrombolytic drugs be avoided as their use has been reported to worse the clinical condition, presumably due to a further extension of dissection.^7^ In this study, however, 5 A-SCD patients received thrombolytic therapy and had good results. Perhaps, thrombolytic drug is harmful for NA-SCD but may be beneficial for A-SCD with STEMI, which needs future studies to confirm.

Several series have reported poor technical success with PCI for NA-SCD. The PCI failure rate was reported 53% in the Mayo Clinic series and 36% in the Vancouver series.^14^,^15^ Interestingly, the PCI success rate was high (94%) in our study. There are two explanations. One is that those PCI patients were selected because their SCD were either short or simple. The other is that most PCI patients had atherosclerosis which was speculated to halt SCD extension during PCI.^16^

In our study, SCD patients with LAD and/or triple-vessel lesions (including dissected and atherosclerotic lesions) tend to be treated with CABG. Follow-up results have showed that CABG as a treatment strategy for SCD is associated with excellent long-term outcomes. However, because of sample size limitation and possible selection bias, definite conclusions on effectiveness of CABG in this clinical setting cannot be drawn. Moreover, no CABG patient in the study receives repeated coronary angiography during follow-up, so the incidence of late bypasses graft occlusion is unclear.

Many series of NA-SCD have a similar long-term prognosis between the revascularization and conservative therapy and a “watchful waiting” strategy is thus recommended for NA-SCD, unless with ongoing or recurrent ischemia.^8^ Unlike these NA-SCD series, our series mainly consists of A-SCD and a better long-term outcome has been found in the revascularization than conservative therapy. This different effectiveness of revascularization between A-SCD and NA-SCD suggests that the revascularization strategy might be more suitable for patients with A-SCD.

### Limitations

There are several limitations in the study. First, as most SCD patients had atherosclerosis, the results cannot be extended to NA-SCD patients. Second, as IVUS and OCT are not routine tests for ACS in our center, non-classic SCD (intramural hematoma) are inevitably missed. Third, this is a non-randomized retrospective research. Thus, selection bias and uncontrolled confounding limit the ability to assess SCD long-term outcome between different treatments.

## CONCLUSIONS

In China, SCD predominantly affects male population and has a relatively high prevalence of vascular risk factors and atherosclerosis. SCD often affects RCA and causes a short dissection. In-hospital mortality rate is low. The long-term prognosis of patients with SCD seems to be favorable in the revascularization versus conservative therapy.
